# Divide and Conquer: A Tailored Solid‐state NMR Approach to Study Large Membrane Protein Complexes

**DOI:** 10.1002/anie.202203319

**Published:** 2022-07-07

**Authors:** ShengQi Xiang, Cecilia Pinto, Marc Baldus

**Affiliations:** ^1^ NMR Spectroscopy Bijvoet Center for Biomolecular Research Utrecht University Padualaan 8 3584 CH Utrecht The Netherlands; ^2^ MOE Key Lab for Cellular Dynamics School of Life Sciences University of Science and Technology of China 96 Jinzhai Road Hefei 230026 Anhui China; ^3^ Current address: Department of Bionanoscience Kavli Institute of Nanoscience Delft University of Technology Van der Maasweg 9 2629 H. Z. Delft The Netherlands

**Keywords:** BAM Complex, Isotopic Labelling, Membrane Protein Complex, NMR Spectroscopy, Proton Detection

## Abstract

Membrane proteins are known to exert many essential biological functions by forming complexes in cell membranes. An example refers to the β‐barrel assembly machinery (BAM), a 200 kDa pentameric complex containing BAM proteins A–E that catalyzes the essential process of protein insertion into the outer membrane of gram‐negative bacteria. While progress has been made in capturing three‐dimensional structural snapshots of the BAM complex, the role of the lipoprotein BamC in the complex assembly in functional lipid bilayers has remained unclear. We have devised a component‐selective preparation scheme to directly study BamC as part of the entire BAM complex in lipid bilayers. Combination with proton‐detected solid‐state NMR methods allowed us to probe the structure, dynamics, and supramolecular topology of full‐length BamC embedded in the entire complex in lipid bilayers. Our approach may help decipher how individual proteins contribute to the dynamic formation and functioning of membrane protein complexes in membranes.

## Graphical abstract

We describe a protein‐selective labelling approach that allowed us to probe a single lipoprotein (BamC) in the pentameric BAM membrane protein complex by proton‐detected solid‐state NMR. Our results generate novel insights into the supramolecular structure of the BAM complex and provide evidence that the three BamC domains exhibit different dynamic properties within the 200 kDa complex embedded in lipid bilayers.

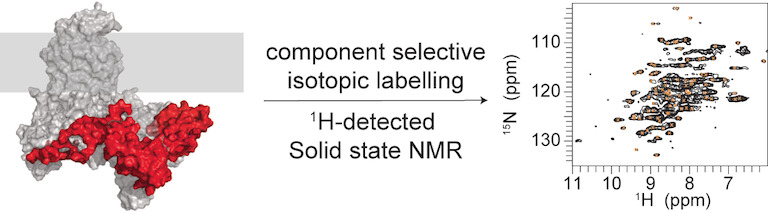

## Introduction

Lipoproteins represent a widespread set of lipid‐anchored proteins involved in essential cellular functions, including maintaining cell shape and biogenesis of the lipid membranes of both gram‐negative^[1]^ and gram‐positive^[2]^ bacteria. In many cases, lipoproteins are involved in signal transduction, cell activity, or the transport of a variety of molecules across the bacterial cell envelope by interactions within larger membrane protein complexes, making them attractive targets for antibiotics and antipathogenic research.

One such complex, the β‐barrel assembly machinery (BAM), is responsible for the insertion and correct folding of outer membrane proteins (OMPs) in gram‐negative bacteria.^[3]^ The BAM protein machinery is essential for physiological, pathogenic, and drug resistance functions^[3a]^ and consists of the central component BamA, and the four lipoproteins BamB, BamC, BamD, and BamE.^[3c]^ While structural and functional information about BamA and the lipoproteins BamB, BamD, and BamE is substantial, structural studies of BamC, which is conserved in BAM complexes of proteobacteria,^[4]^ have remained challenging.

The structure of BamC, which consists of an N‐terminally unstructured region followed by two C‐terminal helix‐grip domains connected by a short linker region, has been solved isolated or when the protein is interacting with other members of the BAM complex.^[5]^ While there is a consensus that the N‐terminal domain of BamC interacts directly with BamD, the interactions between the helix‐grip domains of BamC with BamD (or any other BAM protein) are diverse.[^5a^, ^5b^] In several of the available structures of the complex, at least the N‐terminal domain and the first helix‐grip domain interact with BamD,^[6]^ with one of the structures (PDB ID 5D0Q) also including the second helix‐grip domain.^[7]^ Furthermore, the supramolecular topology of BamC in the lipid bilayer remains a topic of controversy, and experimental evidence suggests that portions of BamC may be surface‐exposed towards the outside of the bacterial cell.^[8]^

Solid‐state NMR (ssNMR) has long been used as a spectroscopic tool to infer membrane protein structure and dynamics in the lipid bilayer.^[9]^ Proton(^1^H)‐detected ssNMR can further increase spectral dispersion and reduce the required sample amount. ^1^H‐detected ssNMR has provided valuable structural^[10]^ and dynamic insight^[11]^ into the workings of membrane proteins, including the dynamics of BamA and the assembly of a 130 kDa BAM subcomplex in lipid bilayers.[^6b^, ^12^] In addition, protein‐protein and protein‐membrane interactions have been examined for small to medium‐sized membrane proteins,[^9d^, ^9e^, ^13^] but studying individual protein components in a large multi‐protein complex, such as the entire 200 kDa BAM complex, has remained challenging.

To tackle the spectroscopic challenges related to dealing with large membrane protein complexes, we have developed an approach to selectively isotope‐label a membrane protein (here BamC) in the context of the entire complex.

## Results and Discussion

**Figure 1:**
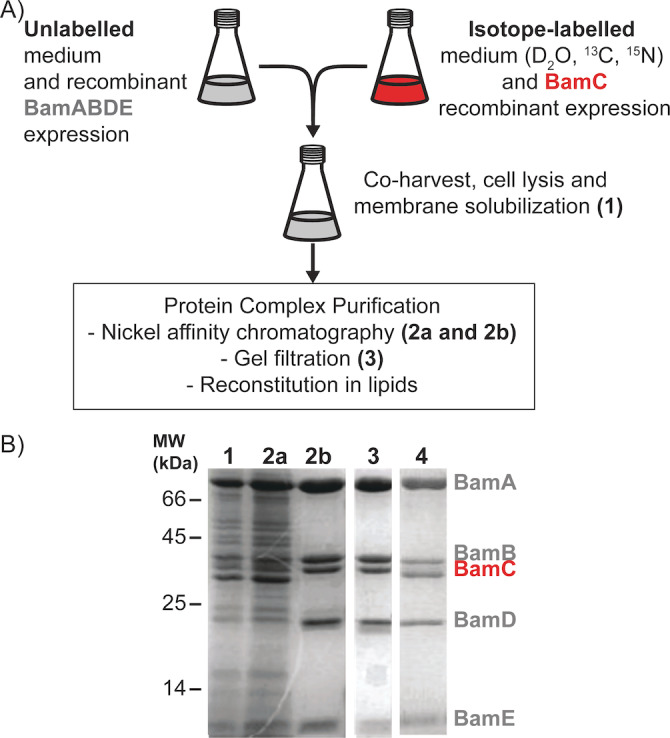
Component (i.e., BamC)‐selective preparation of uniformly (^2^H, ^13^C, ^15^N) labeled BamC in complex with unlabeled BamABDE. A) Scheme for targeted labeling of the BamC lipoprotein and BamABDE unlabeled complex members. For a more detailed description of sample preparation, please see the methods in Supporting Information. B) SDS‐PAGE analysis of BamABCDE sample preparation throughout the purification process. Lane 1. Detergent solubilized fraction of combined BamABDE and BamC over‐expressing cells. Lanes 2a and 2b, unbound and eluted fractions, respectively, of the nickel affinity purification. Lane 3. Resulting BamABCDE complex after size exclusion chromatography. Lane 4. Purified BamABCDE complex was obtained from the expression of these genes under one promoter.

**Figure 2:**
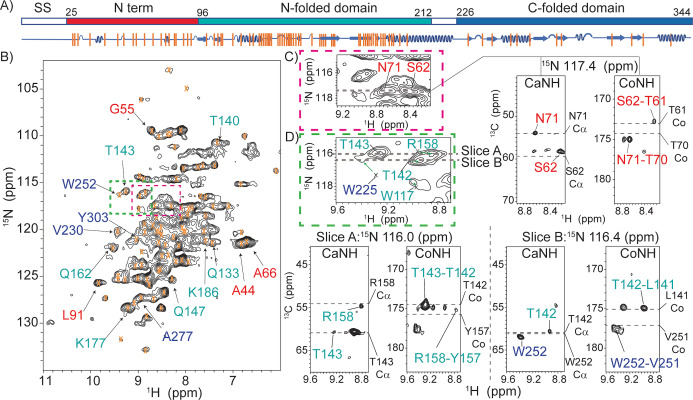
^1^H‐detected solid‐state NMR spectra and the transferred assignments for BamC within the membrane‐embedded BAM complex A) Schematic diagram of the BamC protein indicating the signal sequence (ss, residues 1–25, white, which is removed during processing), N‐terminus (residues 25–96, red) as well as the N‐folded domain (residues 97–212, cyan) and C‐folded domain (residues 226–344, blue). The secondary structure elements are shown below the diagram in blue. Orange bars represent assigned residues. B) 2D proton‐detected NH correlation spectrum of BamABCDE. Orange crosses represent transferred BamC assignments as described in the text. Correlations follow the domain coloring given in (A). Green and red colored rectangles indicated in B are analyzed by 3D ssNMR in Figures C and D. C) Examples for the N‐terminal domain. The dashed lines in the 2D NH and 3D CANH/CONH strips show the predicted N, Cα, and Co chemical shifts. D) Examples of residues identified for the two folded domains. Again, the dashed lines in the 3D trips show the chemical shifts of Cα and Co obtained from solution‐state results. All spectra were recorded at a sample temperature of 303 K.

For this purpose, we made use of the fact that lipoproteins, such as BamB–E, are inserted into the outer membrane (OM) of *E. coli* via a general (and thus BAM‐independent) mechanism, known as the Lol pathway.^[14]^ We designed a protocol for the independent expression of the target protein (BamC) and the remaining components (BamABDE) of the entire complex. After recombinant protein expression in the desired medium, care was taken to ensure that sufficient BamC expressing cells were added to saturate the BamABDE subcomplex. Cell cultures were combined prior to cell lysis and membrane solubilization (Figure 1A, B lane 1) due to the extended random coil region (N‐terminus, residues 25–95, Figure 2A) of BamC making this protein prone to degradation in the absence of complex members, namely BamD. Because the histidine tag is present on BamE, nickel affinity chromatography removed not only contaminating proteins and endogenous BAM complexes containing labelled BamC but also any excess, unincorporated BamC, or incompletely assembled BAM complexes (Figure 1B, lanes 2a and 2b). The BAM complex obtained via this methodology, hereby referred to as BamABCDE (Figure 1B, lane 3), contained isotope‐labeled BamC incorporated at comparable levels to those when all complex members are expressed under one promoter (Figure 1B lane 4). Furthermore, size‐exclusion chromatography of the BamABCDE complex indicated that the sample does not contain aggregates, highlighting the stability of the complex and allowing us to produce homogeneous and well‐folded samples (Figure S1).

This 200 kDa BamABCDE complex was reconstituted in lipid bilayers and subjected to proton‐detected ssNMR experiments. Moreover, we relied on dipolar‐(cross polarization, CP) based ssNMR spectroscopy at elevated (303 K) temperatures, where rigid protein segments remain visible, but ssNMR signals of protein regions that exhibit enhanced dynamics disappear (see, e.g., Ref. [15]). 2D [^1^H, ^15^N]‐correlation spectra obtained on an 800 MHz instrument at 55 kHz MAS and a sample temperature of 303 K (Figure 2B) showed good resolution, even though only BamC was deuterated in the entire complex. Subsequently, we acquired 3D CANH and CONH data for one week of signal accumulation (Figure 2C, D and Supporting Information). The use of pulse sequences that require further magnetization transfer steps, such as CacoNH, leading to sequential assignments,^[16]^ was prohibited due to insufficient sensitivity.

Instead, we applied a tailored ssNMR analysis that makes use of NMR data obtained on the soluble components of the protein of interest (BamC) and structural evidence from X‐ray/cryoEM data on BAM (sub)complexes. Besides the BAM complex structures, structural information on BamC is also available for the N‐terminal flexible domain (Figure 2A, red) bound to BamD.^[5a]^ In addition, the two helix‐grip domains (denoted N‐folded in cyan and C‐folded, blue, in Figure 2A, respectively) have been determined individually and as a whole.[^5a^, ^5c^, ^5d^]

We analyzed the 2D and 3D ^1^H‐detected ssNMR spectra using solution NMR assignments obtained for an isolated (soluble) BamC construct (BMRB 16035, Ref. [17]) as a reference. In addition, the chemical shifts of the N‐terminal flexible domain of BamC were calculated using SHIFTX2^[18]^ and the X‐ray structure of the complex (PDB 5D0Q, Ref. [7]). Chemical shift values predicted on the basis of these reference data were considered as valid assignments of BamC as part of complex if all three frequencies (^15^N,^13^Cα, and^13^Co) in the 3D CaNH and CoNH ^1^H ssNMR spectra matched within 1.5 ppm.^[19]^ (Figure 2C, D and S2).

**Figure 3:**
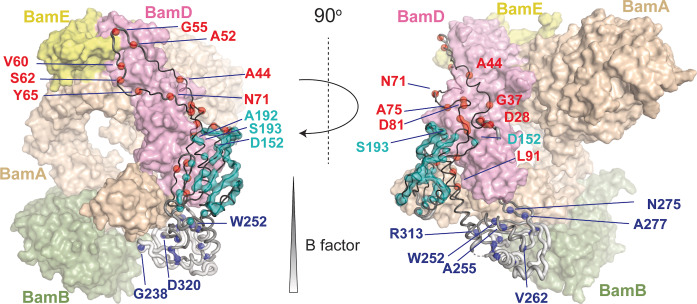
Assigned BamC residues in the BAM complex. BamC structure from PDB 5D0Q with assigned BamC residues indicated in spheres and colored as in Figure 2. BamABDE proteins are represented as transparent surfaces with the indicated colors of each BAM protein for reference. Some BamC residues that are close to the protein interfaces are labeled. The entire list of assigned residues is given in the Supporting Information. The radius of the BamC backbone was scaled by its residue‐specific B factor value using the Pymol “B factor putty” function^[22]^ and was colored from black (low B factor) to white (high B factor). We attribute the absence of signals in the putative α‐helix between the two folded domains of BamC to protein mobility.

Importantly, and unlike previously,^[20]^ only the protein of interest (BamC) is labeled, and the existing solution NMR and predicted assignments cover the entirety of the protein. In addition, the enhanced spectral resolution in our 3D ^1^H ssNMR data sets (Figure 2C and D) allowed us to identify several amino‐acid stretches within all three structurally defined regions (Figure 2A, orange bars, and Figure 3 residues in spheres).

In total, we obtained Cα, Co, N, and H^N^ chemical shifts for 86 residues. We also included residues A52, A54, and V60 in the N‐terminal region exhibiting slightly larger deviations which we attribute to limitations of the prediction software due to neighbouring proline residues. In addition, we could identify 2 residues (W117, G138) for which the chemical shifts matched with previous solution‐state NMR data in 2 frequencies (N and H^N^). Vertical orange lines in Figure 2A label these residues, and the chemical shifts are listed in Supporting Information Table S3. Example correlations from all domains of the protein (N‐terminus and the two folded domains, Figure 2A) that are well resolved in the 2D and 3D spectra are given in Figure 2B as well as Figure 2C/D, respectively.

We assigned 20 residues (out of the 71) in the N‐terminal domain of BamC (Figure 2 and 3, red) that tightly packs against BamD in the available X‐ray structures. As visible in Figure 3, these residues almost fully cover the binding epitope seen in crystals, indicating that this interface also exists after reconstitution in lipid bilayers. Notably, the first residue we observed was D28, implying that the entire N‐terminal domain is involved in protein‐protein interactions. We could also assign nearly half (57 residues out of 116) of the N‐folded (helix‐grip) domain of BamC (Figure 2 and 3, cyan). When plotted on the 3D structure reported for the BAM complex (PDB ID 5D0Q, Figure 3), these assigned residues cover a large part of the entire N‐folded domain. In the BamD binding region, we could identify a number of BamC residues (D152, A192, and S193), suggesting that this domain is folded properly and forms a tight complex with BamD (Figure 3). In contrast, we could only observe 11 out of 118 residues of the C‐folded BamC domain (Figure 2 and 3, blue) in our ssNMR data probing rigid protein segments. Most of these residues, including G238, W252, and D320, are located close to the binding interface with BamA.

In general, missing assignments in our ssNMR analysis could result from structural variations that induce chemical shift changes and/or they may be due to increased dynamics that lead to signal loss in our dipolar‐based experiments. Visual inspection of our 2D data set (Figure 2B) already indicated that not all 344 residues of BamC contribute to the dipolar ssNMR spectrum at 303 K. Moreover, a detailed analysis of our 3D data spoke against a significant population of correlations that deviated from the structural model shown in Figure 3. Even in the most crowded region, most peaks could be readily assigned (Figure S3), suggesting that the number of protein residues that remain unaccounted for in our current analysis is small. Hence, we consider increased dynamics as the dominating effect for residues absent in our ssNMR analysis at 303 K.

While the N‐folded domain (Figure 2 and 3, cyan) is elusive in roughly half of all known BAM complex structures, our ssNMR data reveal that this domain is the most well‐defined protein domain of BamC in our bilayer preparations. This N‐folded domain is in contact with BamD and BamA via the N‐terminal region, limiting any large‐scale motions. Moreover, the assigned residues in the N‐folded domain are primarily located in the β sheets forming the core. The inter‐strand contacts may further stabilize the backbone, thus reducing local fluctuations.

In addition, the epitope that defines the binding between the N‐terminal domain (Figure 2 and 3, red) and BamD seems to be largely conserved in our bilayer preparations (Figure 3). However, residue stretches such as Y31‐G37 or A44‐L50 that could not be identified may undergo enhanced dynamics leading to the disappearance of these ssNMR signals in our data. Indeed, previous molecular dynamics simulations predicted such significant residue‐specific dynamics in the N‐terminal domain in both the entire complex and the BamACDE subcomplex.^[7]^

The C‐folded BamC domain (Figure 2 and 3, blue) has remained elusive in most BAM complex structures with a few exceptions.[^6^, ^7^, ^21^] Even if this domain presents in the reported structures, its B factor values are significantly higher than in the rest of the BamC protein (Figure 3). Our solid‐state NMR data suggest that this domain indeed exhibits elevated dynamics compared to the rest of the protein, significantly reducing the number of ssNMR correlations seen in our dipolar spectra. This notion is also in agreement with previous molecular dynamics simulations suggesting the helix linking two folded domains and the C‐folded domain displayed significant fluctuations.^[7]^ Because the two folded domains exhibit different dynamic properties, it is unlikely that the linker connecting them (residues 213—226) forms a stable helix. This notion is consistent with their absence of signals in our dipolar spectra and a previous solution‐state NMR study showing that this region is not a well‐folded helix but rather exhibits α‐helical propensity^[17]^.

**Figure 4:**
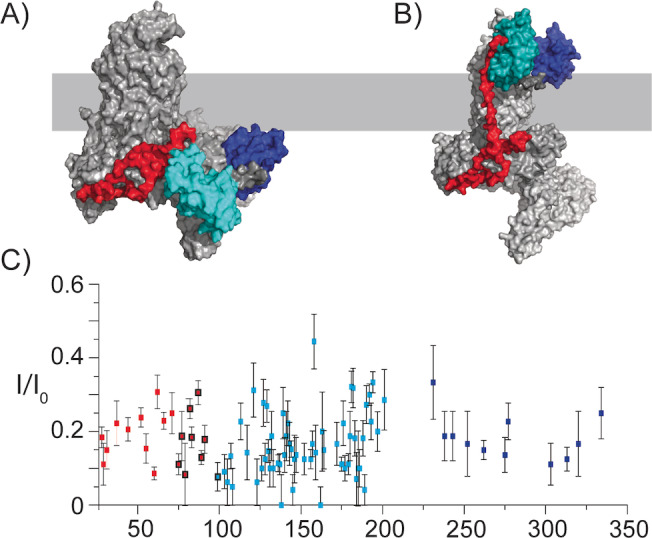
PRE profile speaks against the surface exposure of BamC in the BAM complex. A) X‐ray structure obtained for the BamABCDE complex in detergent (PDB 5D0Q). B) The theoretical model constructed based on the study of O'Neil et al.^[24]^ for the BamC topology, where the folded domains are exposed to the cell surface. For (A) and (B), the approximate positions of the membrane are depicted in light grey. The domains of BamC are colored as indicated in Figures 2 and 3, and all other BAM complex members are in grey. C) PRE effect of a paramagnetic agent (Gadolinium DOTA) on ssNMR intensities of the assigned residues of BamC (Table S4). Error bars were estimated based on the S/N ratios of each residue. Residues 75 to 100 are in the N‐terminal region and are highlighted by black circles, indicating the putative membrane‐traversing region.

Subsequently, we probed the supramolecular structure of BamABCDE in the lipid bilayer. In particular, we examined whether the observed ssNMR data would provide insight into a potential surface exposure of BamC in the context of the entire BAM complex.^[8]^ Unlike the case seen in the X‐ray structures (Figure 4A), such a surface exposure would require a membrane‐spanning region in the N‐terminal domain of BamC, as indicated in Figure 4B. If such as transmembrane region would form a β‐strand conformation, at least 9 amino acids would be required to traverse the outer membrane (OM) (hydrophobic thickness of ≈24 Å^[23]^), while at least 17 amino acids would need to adopt an α‐helical conformation across the OM. Within the putative transmembrane part (roughly comprising residues 74–102), we identified 9 residues. Their measured chemical shifts (Figure 2A and Figure S2A, red domain) match well with predicted values from the known X‐ray structures and would be less consistent with the topological model of O'Neil et al.^[24]^ (Figure 4B and Figure S2B). We also note that these values would be inconsistent with either α‐helix or β‐strand transmembrane elements. To gain further insight into the overall topology of BamC, we added a paramagnetic agent (Gd^3+^) to our proteoliposome preparations and examined the resulting signal decay due to paramagnetic relaxation enhancement effects.^[25]^ For all assigned ssNMR residues, including those in the proposed OM traversing region, we observed a reduction in the intensity of at least 65 % (Figure 4C, Table S4). These findings suggest that at least the assigned residues, including those of the putative membrane‐spanning N‐terminal segment, experience an increased relaxation rate due to the presence of the paramagnetic agent within ≈15 Å.

Together with the data presented in Figures 2 and 3 and our earlier observations that BamA inserts in a uniform orientation into membranes,^[26]^ these results would favor a supramolecular fold without a transmembrane‐spanning (N‐terminal) region of BamC in our reconstituted bilayer preparations.

## Conclusion

In summary, our component‐selective labeling approach allowed us to study structural and dynamical aspects as well as the supramolecular fold of BamC as part of the entire 200 kDa BAM complex in lipid bilayers. Our data are in agreement with the binding epitopes of the N‐terminal and N‐folded domain of BamC to BamD and BamA seen in some crystal structures.[^6^, ^7^, ^21^, ^27^] The PRE results furthermore support the notion that BamC does not traverse the membrane as suggested in other studies^[8]^ and are in line with recent EM experiments using nanodiscs.^[28]^ On the other hand, we find spectroscopic evidence that the C‐terminal fold domain but also segments of the N‐terminal domain exhibit significant dynamics in qualitative agreement with previous MD studies and elevated B factors. Since the chemical shifts of residues identified in the C‐folded domain match with those obtained in free solution, the dynamic profile may involve both residue‐specific local as well as global domain (e.g. on/off binding) motion. Such dynamics would be in line with the missing densities in the X‐ray and EM structures[^6^, ^7^, ^21^, ^28^] and may, similar to residue‐specific motions in BamA,[^12^, ^28^] be critically needed during the protein insertion cycle of the BAM complex.

Combining our component‐selective labelling approach with the increasing sensitivity of ultra‐high field ^1^H ssNMR may allow to further dissect such dynamic processes. In addition, our preparatory scheme can be adapted for other spectroscopic methods such as EPR^[29]^ or readily combined with advanced computational predictions,^[30]^ possibly leading to unprecedented insights into how membrane protein complexes are assembled and function in a dynamic cell membrane setting.

## Conflict of interest

The authors declare no conflict of interest.

1

## Supporting information

As a service to our authors and readers, this journal provides supporting information supplied by the authors. Such materials are peer reviewed and may be re‐organized for online delivery, but are not copy‐edited or typeset. Technical support issues arising from supporting information (other than missing files) should be addressed to the authors.

Supporting Information

## Data Availability

The data that support the findings of this study are available from the corresponding author upon reasonable request.
