# Supplementary material for: Divide and Conquer: A Tailored Solid‐state NMR Approach to Study Large Membrane Protein Complexes
Source: Angew Chem Int Ed Engl. 2022 Jul 7;61(33):e202203319. doi: 10.1002/anie.202203319 (PMC9540533; doi:10.1002/anie.202203319)
Supplement: Supplementary file 1 — Supporting Information [file ANIE-61-0-s001.pdf]

Angewandte Chemie
Eine Zeitschrift der Gesellschaft Deutscher Chemiker

Supporting Information

Divide and Conquer: A Tailored Solid-state NMR Approach to Study Large Membrane Protein Complexes

S. Xiang, C. Pinto, M. Baldus**

Supplementary Information

This PDF file includes:

Supplementary text
Figures S1 to S3
Tables S1 to S4
SI References

Supplementary Information Text

Extended Methods

BamAB<u>C</u>DE sample preparation

Cloning

We modified the pJH114 plasmid in order to obtain independent plasmids for the expression of the BamABDE complex and BamC protein as described below.

pBAD_BamABCDE plasmid

The promoter region of the pJH114 plasmid was exchanged with that of the pBAD plasmid. Amplification of the promotor region of the pBAD vector (3188-294) included the introduction of restriction sites ApaI (3188) and NdeI (294) using the primers FW_pBAD (5'-ATA ATA GGG GCC CTT ATG ACA ACT TGA CGG CTA CAT C-3') and RV_pBAD (5'-ATA ATA CCA TAT GAA AAC GGG TAT GGA GAA ACA GTA-3'). The resulting PCR reaction was treated with DpnI at 37°C for an hour prior to purification with Agencourt AMPure XP (Beckman Coulter). In order to introduce the new promotor, pJH114 must be treated with NdeI and ApaI (this removes the LacQ and Trc promoter region) and then ligate the PCR product in the vector. The vector was gel purified, briefly, 2 µL loading buffer was added to the 15 µL restriction reaction and run on a 0.8 % agarose gel for 2 h. The band corresponding to the linearized plasmid was excised, and DNA was extracted with a Qiagen gel extraction kit. The pBAD_BamABCDE vector was obtained by ligation of the obtained pBAD promotor gene into the NdeI and ApaI pre-treated pJH114 vector. After 5 h incubation at 37°C, 1 µl CIAP, T4 ligase was added and incubated for a further 1 h before 10 min inactivation at 65°C for 10 min.

pBAD_BamABDE plasmid

The pBAD_BamABDE plasmid was obtained by removal of the bamC gene from the pBAD_BamABCDE plasmid. NarI restriction sites were introduced by mutagenesis at the N- and C- terminus of the bamC gene within the plasmid pBAD_BamABCDE as a template and primers FW_C_Nter (5'- ACT CTA TTA CAC GTT AAT CGG CGC CTA GGG AGA TTT GAT GGC TT-3') and RV_C_Cter (5'- GCT GCG TTT AGC AAG TAA GGC GCC TGA GGA AAG TCA AAA CGT-3'). The resulting PCR reaction was treated with DpnI at 37°C for an hour prior to treatment with NarI to remove the bamC gene. The resulting linear vector was purified by gel extraction. Briefly, 2 µL loading buffer was added to the 15 µL restriction reaction and run on a 0.8% agarose gel for 2 h. The band corresponding to the linearized plasmid was excised and DNA was extracted with a Qiagen gel extraction kit. The pBAD_BamABDE vector was obtained by ligation of the obtained NarI pre-treated pBAD_BamABCDE vector. After 5h incubation at 37°C, 1µl CIAP, T4 ligase was added and incubated for a further 1 h before 10 min inactivation at 65°C for 10 min.

The resulting mixtures were transformed into *E. coli* DH5α cells for plasmid preparation and all DNA sequences were confirmed by sequencing.

Protein expression, purification and reconstitution into liposomes

a. Unlabeled BamABDE

For the BamABDE complex and BamC, E. coli Lemo21(DE3) cells were transformed with the pBAD_BamABDE plasmid and plated on LB/agar plates supplemented with chloramphenicol and ampicillin, as well as 0.4% glucose. Minimum M9 medium cultures with glycerol (5g/L) instead of glucose were inoculated and grown at 37°C to an optical density of 0.8 after which they were induced with 2 g/L arabinose and incubated for 3.5 h at 37°C.

b. Fractional deuteration of ^{13}C, ^{15}N labeled BamC

For expression of the BamC protein, E. coli Lemo21(DE3) cells were transformed with the pCDF_BamC plasmid. Deuterated minimum M9 medium (99% D2O) cultures supplemented with ^{13}C glucose and ^{15}N NH_4Cl were inoculated and grown at 37°C to an optical density of ~0.7, after which they were induced with 0.5 mM IPTG. Cultures were grown for an additional 7h at 37°C. Cultures of BamABDE and BamC overexpressing cells were combined in a 4:1L ratio to ensure saturation of BamABDE complexes and harvested jointly (4 000xg, 4°C, 20 min). The resulting cell pellet was washed with PBS and frozen at -20°C.

Complex purification and reconstitution into liposomes

The cell pellet was washed with 50 mM Sodium phosphate pH 8.0, 150 mM NaCl, 10 mM imidazole, 1 mM β-mercaptoethanol, proteinase inhibitors, and lysozyme. Lysis was achieved by multiple passes through a French press (3x 10 Kpsi) followed by centrifugation at 4 000xg for 15 min at 4°C. The supernatant was ultracentrifuged at 80 000xg for 45 min at 4°C. The resulting membrane pellet was dissolved in 50 mL 50 mM Sodium phosphate pH 8.0, 150 mM NaCl, 10 mM imidazole, 1% n-Dodecyl β-D-maltoside (DDM, Avanti), proteinase inhibitors and incubated for 3h at 4°C. The solubilized membranes were ultracentrifuged for 45 min at 80 000xg for at 4°C and subsequently filtered before addition to Ni-NTA agarose beads (Qiagen) and incubated overnight at 4°C. The Ni-NTA beads were washed with 20 column volumes of 50 mM Sodium phosphate pH 8.0, 150 mM NaCl, 25 mM imidazole, 0.03% DDM. Elution was achieved with 4 column volumes of 50 M Sodium phosphate pH 8.0, 150 mM NaCl, 0.3 M imidazole and 0.03% DDM. The sample was concentrated with an Amicon ultra-15 centrifugal unit (50 kDa cut-off, Millipore Sigma) before application to a pre-equilibrated Superdex 200 16/60 (GE Life Sciences), with 50 mM Sodium phosphate, 0.1 M NaCl, 0.5 mM ß-mercaptoethanol, 0.03% DDM and run on this column at 1 ml/min. The resulting fractions were pooled, and concentration was determined with the measurement of the absorbance at 280nm on a nanodrop. The BamABCDE complex was reconstituted with DLPC lipids at a 100:1 mol/mol lipid-to-protein ratio via dialysis against 20 mM Sodium phosphate, pH 7.0 at 4ºC for approximately a week with multiple changes to the dialysis buffer. Liposomes were harvested at 4 000xg at 4°C for 30 min. If the sample was treated with a paramagnetic agent, the liposomes were additionally washed with 10 mM gadodiamide (Omniscan, GE Healthcare). The resulting liposome pellet was packed into a 1.3 mm rotor (Bruker Biospin) prior to the measurements.

Solid-State NMR experiments

The proton-detected NMR experiments of [^2H, ^{13}C, ^{15}N] labeled BamABCDE samples were carried out at 55kHz on a wide bore 800MHz spectrometer equipped with a 1.3 mm HXY MAS probe (Bruker Biospin). The actual sample temperature was 303K at 55kHz spinning, calibrated by an external DSS standard sample. The 2D NH, 3D CaNH, and CoNH experiments were implemented as described previously[1]. The parameters for each transfer step and acquisition are listed in Tables S1 and S2, respectively. Each 3D spectrum for BamABCDE was recorded in approximately one week of measurement time by adding data sets of 1-day length. During the measurement periods, the magnetic field drifting was monitored by the H_2O signal positions and compensated later. The PRE effects are calculated as the peaks intensities ratios on the 2D NH spectra of Gd3+ doped and reference sample and normalized by the number of scans of each spectrum.

Supplementary Information Figures

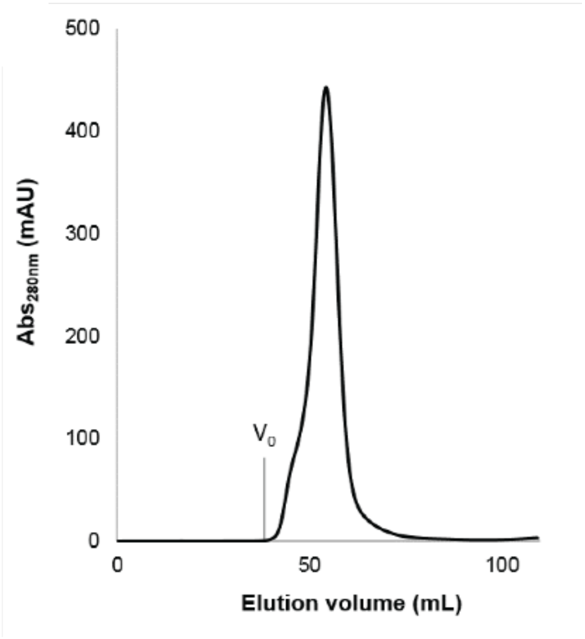

Fig. S1. size-exclusion chromatography of the BamABCDE complex. Representative size exclusion chromatography curve obtained for the BamABCDE complex in detergent on an S200 16/60 PG column. The main peak (50-70 mL) was utilized for subsequent sample preparation, displaying the homogeneity of a correctly formed and well-behaving complex, eluting at a volume identical to the BamABCDE complex (~52 mL).

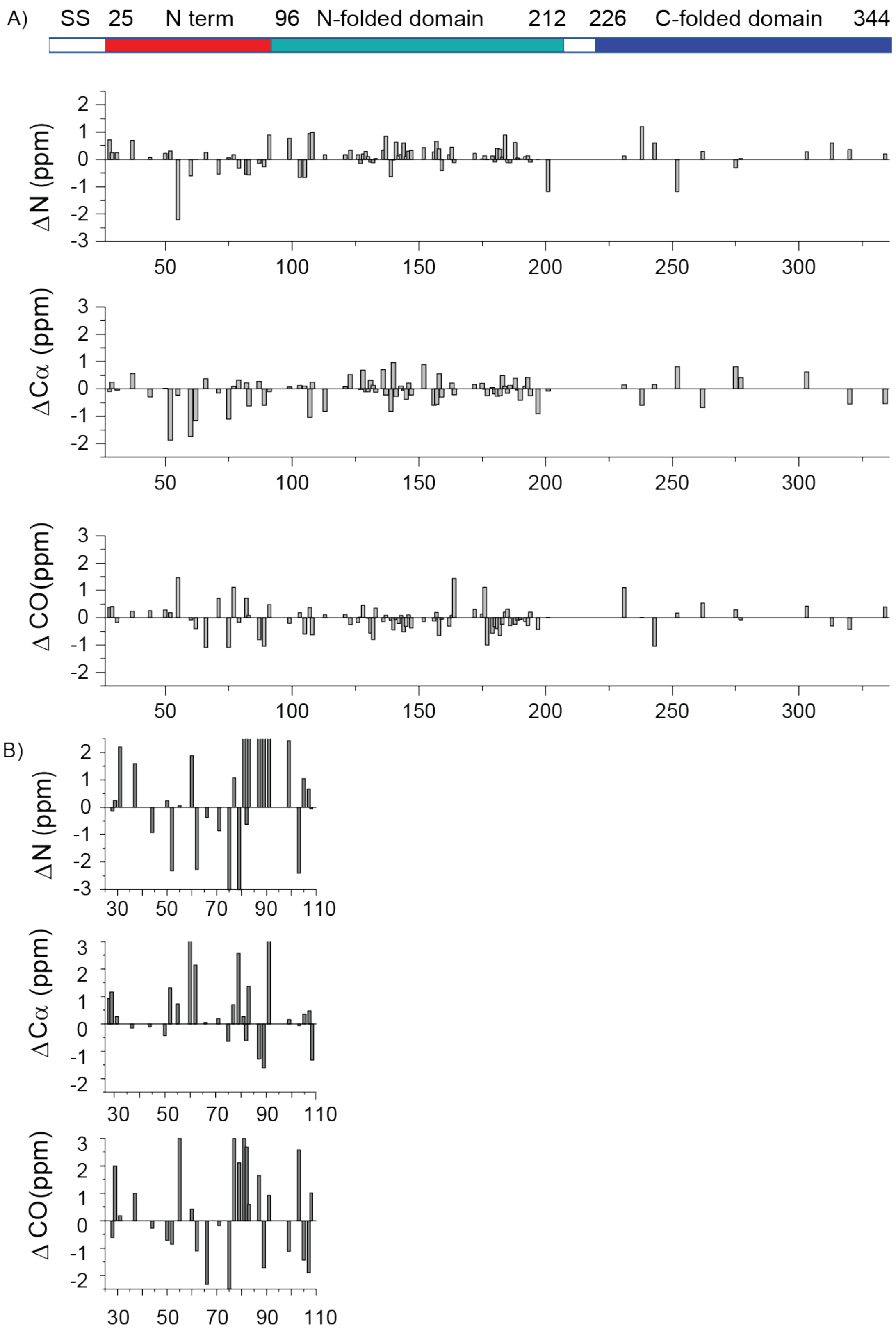

Fig. S2. BamC chemical-shift analysis. The residue-specific difference in chemical shifts obtained in the current solid-state NMR study and previous results. A) Chemical-shift values for the two

folded domains were taken from solution-state NMR assignments (BMRB 16035), while the N-terminal chemical shifts are predicted from the BamABCDE X-ray structure (PDB 5D0Q). B) The chemical shifts difference between solid-state NMR results and the prediction values based on the transmembrane model from the reference[2].

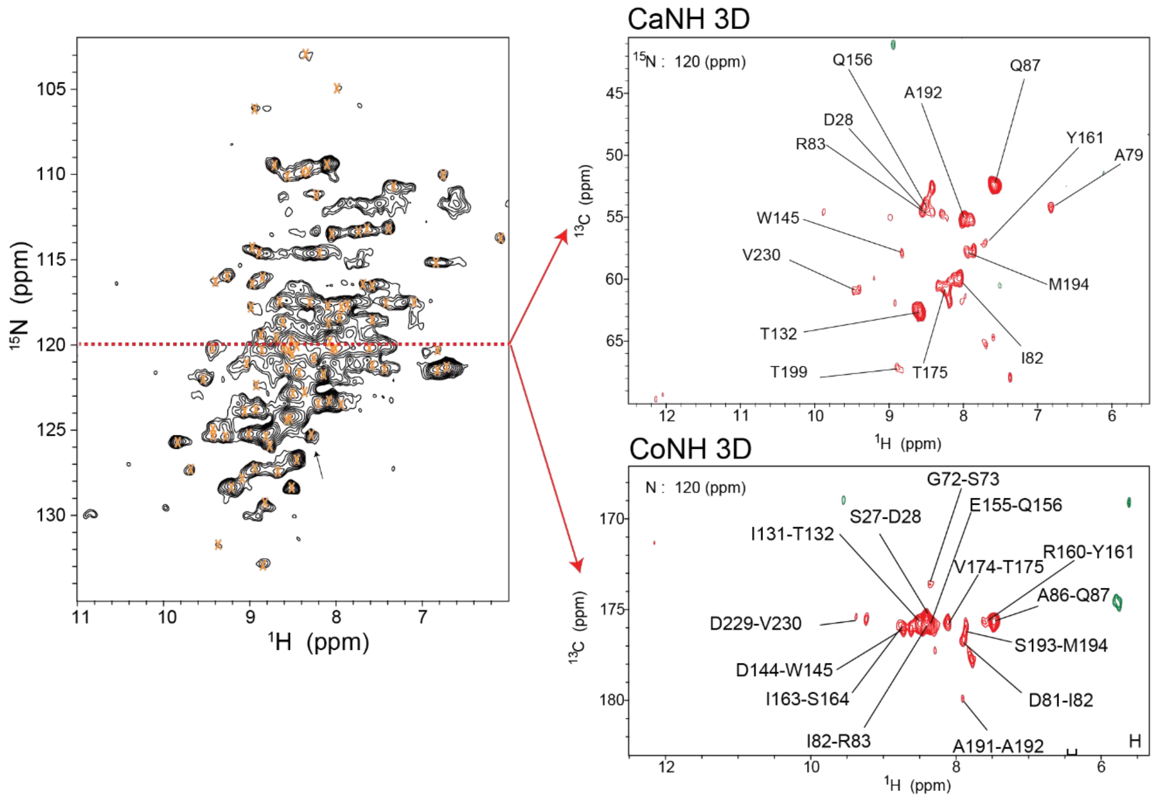

Fig. S3. Cross-sections of 3D ^{1}H-detected spectra of the BAMAB<u>C</u>DE complex. 2D planes of 3D CaNH and 3D CoNH are shown at the most crowded region, as indicated by the red dash line on 2D NH. The assigned peaks were labeled.

Supplementary Information Tables

Transfer steps	Contact time(μs)	Pulses
H->Cα	3500	H:93.5 kHz (90-100 % ramp on H)/C:43.5 kHz
H->Co	3500	H: 93.5 kHz (90-100 % ramp on H)/C:20.3 kHz
H->N	1400	H:105.3kHz (80-100 % ramp on H)/N:45.5kHz
C'->N	6000	N:18.3kHz/C': 37.6 kHz (100-90 % ramp on C')
Cα->N	6000	N:18.3 kHz/Cα:37.6kHz (100-90 % ramp on Cα)
N->H^N	600	H:91.7 kHz (100-90 % ramp on H)/N:45.5 kHz

Table S1: experimental parameters for the dipolar transfers of ssNMR experiments of BamABCDE at 55 kHz.

Spectrum	Nucleus	Spectral width (Hz)	Maximum acquisition time (ms)
2D NH	N	3244	20
	H^N	16025	20
3D CANH	Cα	6037	2.3
	N	2676	6.0
	H^N	16025	20.0
3D CONH	CO	3220	3.1
	N	2676	6.0
	H^N	16025	20.0

Table S2: acquisition parameters for the proton detected ssNMR spectra

Res.	N(i)	H^N(i)	Cα(i)	Co(i-1)	Res.	N(i)	H^N(i)	Cα(i)	Co(i-1)
D28	120.3	8.56	54.6	175.5	T143	115.9	9.26	60.7	174.7
S29	114.6	8.19	58.4	176.7	D144	118.4	7.96	52.2	174.9
Y31	118.7	8.09	60.2	176.8	W145	120.3	8.86	57.9	176.1
G37	109.8	8.32	44.8	175.0	V146	127.3	9.69	61.7	176.8
A44	121.5	6.82	52.0	177.0	Q147	125.3	8.29	54.3	174.5
L50	124.0	8.10	53.8	176.1	D152	116.5	7.58	54.6	177.3
A52	125.3	8.81	48.8	174.9	Q156	119.7	8.52	54.4	175.5
G55	109.4	8.71	45.1	179.2	Y157	122.8	8.50	56.4	176.3
V60	123.3	8.08	63.2	176.4	R158	116.0	8.85	54.6	175.2
S62	117.5	8.31	58.2	172.7	G159	106.1	8.95	44.1	175.0
A66	121.3	6.72	52.0	174.3	Q162	122.0	9.54	54.2	173.7
N71	117.4	8.66	53.9	175.0	I163	131.7	9.37	59.8	175.9
A75	121.6	8.43	51.2	172.0	S164	119.4	8.87	56.0	175.8
G77	110.1	8.56	45.2	177.6	Q172	118.6	8.62	54.7	175.2
A79	120.3	6.82	54.2	177.2	T175	120.8	8.26	61.0	175.8
I82	119.8	8.07	60.1	176.7	V176	125.3	9.42	61.0	174.6
R83	120.5	8.51	54.8	175.8	K177	128.3	9.21	53.7	172.1
Q87	120.2	7.61	52.4	175.7	L179	126.7	8.46	55.8	174.0
L89	121.7	8.14	53.1	174.7	N180	110.0	6.76	53.3	177.1
L91	125.7	9.84	57.1	177.5	L181	125.2	9.01	53.7	171.2
F99	123.8	8.93	56.4	173.5	E182	125.3	9.28	53.7	173.9
T103	113.3	7.74	61.5	175.6	Q183	121.4	8.64	54.9	174.3
S105	114.3	8.97	57.1	174.8	A184	132.9	8.85	52.8	175.7
L107	128.4	8.52	53.0	175.0	G185	102.9	8.35	44.7	177.5
V108	123.9	9.06	60.9	175.9	K186	121.2	7.58	52.6	173.4
G113	109.8	8.38	45.0	177.6	V188	122.8	8.36	61.7	176.8
W117	117.8	8.99	N.A.	N.A.	A189	125.9	8.77	50.9	175.0
V121	117.5	7.42	66.9	177.3	D190	118.5	7.58	53.5	176.6
V123	123.5	7.94	66.1	177.2	A192	120.2	8.04	55.2	179.9
A126	123.4	8.21	54.1	180.5	S193	117.7	8.09	62.4	181.3
K127	115.2	6.84	53.7	179.5	M194	120.2	8.01	57.8	176.2
N128	113.5	8.05	54.6	176.0	Y197	113.2	7.64	61.9	178.4
Y129	117.8	7.94	54.1	175.0	M201	113.7	6.09	56.2	178.6
T130	120.2	8.61	62.7	175.8	Q231	122.4	8.93	53.7	175.4
I131	129.3	8.82	60.7	173.0	G238	111.2	8.23	44.1	174.8
T132	119.5	8.69	62.6	175.7	V243	120.2	9.43	60.9	175.5
Q133	121.4	7.44	55.3	175.3	W252	116.3	9.40	62.0	177.4
D136	124.3	8.53	58.1	176.1	V262	109.4	8.11	58.2	177.0
A137	124.4	8.57	54.1	178.0	N275	114.6	8.89	53.1	173.0
G138	104.9	7.99	N.A.	N.A.	A277	127.3	8.94	52.0	173.2
Q139	117.5	7.09	54.3	172.6	Y303	121.1	9.04	57.6	175.8
T140	110.7	7.33	61.4	172.0	R313	117.6	7.87	54.2	173.1
L141	124.9	9.42	53.8	171.7	D320	127.8	9.09	52.3	175.1
T142	116.4	8.97	60.6	174.9	L334	116.4	7.69	54.7	179.5

Table S3: assignments of BamC within BamABCDE complex in the liposome. Please note the Co chemical shifts are of the previous residues.

Res.	Normalized Intensity ratios	Error	Res.	Normalized Intensity ratios	Error
D28	0.18	0.03	T143	0.17	0.06
S29	0.11	0.06	D144	0.15	0.02
Y31	0.15	0.05	W145	0.04	0.04
G37	0.22	0.06	V146	0.13	0.06
A44	0.21	0.03	Q147	0.14	0.05
L50	0.10	0.03	D152	0.13	0.04
A52	0.24	0.03	Q156	0.13	0.03
G55	0.15	0.04	Y157	0.17	0.04
V60	0.09	0.02	R158	0.44	0.07
S62	0.31	0.05	G159	0.14	0.07
A66	0.23	0.02	Q162	0.00	0.05
N71	0.25	0.06	I163	0.20	0.11
A75	0.11	0.03	S164	0.15	0.05
G77	0.19	0.07	Q172	0.17	0.06
A79	0.08	0.08	T175	0.11	0.02
I82	0.26	0.03	V176	0.22	0.06
R83	0.18	0.03	K177	0.10	0.03
Q87	0.31	0.03	L179	0.11	0.03
L89	0.13	0.02	N180	0.19	0.07
L91	0.18	0.04	L181	0.32	0.04
F99	0.08	0.04	E182	0.32	0.05
T103	0.09	0.05	Q183	0.18	0.05
S105	0.06	0.06	A184	0.07	0.07
L107	0.13	0.03	G185	0.10	0.10
V108	0.05	0.05	K186	0.10	0.05
G113	0.23	0.05	V188	0.18	0.05
W117	0.14	0.07	A189	0.04	0.04
V121	0.31	0.07	D190	0.27	0.05
V123	0.06	0.06	A192	0.30	0.03
A126	0.10	0.03	S193	0.23	0.05
K127	0.28	0.06	M194	0.33	0.03
N128	0.13	0.04	Y197	0.20	0.05
Y129	0.27	0.04	M201	0.29	0.08
T130	0.15	0.03	Q231	0.33	0.10
I131	0.10	0.05	G238	0.19	0.07
T132	0.19	0.07	V243	0.19	0.07
Q133	0.10	0.05	W252	0.17	0.09
D136	0.11	0.02	V262	0.15	0.03
A137	0.11	0.03	N275	0.14	0.05
G138	0.00	0.08	A277	0.23	0.05
Q139	0.25	0.07	Y303	0.11	0.06
T140	0.14	0.05	R313	0.13	0.03
L141	0.19	0.07	D320	0.17	0.09
T142	0.22	0.06	L334	0.25	0.07

Table S4: PRE effects of BamABCDE complex in the liposome. The errors were calculated based on the S/N ratios of peaks.

Supplementary Information References

[1] D. H. Zhou, A. J. Nieuwkoop, D. A. Berthold, G. Comellas, L. J. Sperling, M. Tang, G. J. Shah, E. J. Brea, L. R. Lemkau, C. M. Rienstra, *J. Biomol. NMR* **2012**, *54*, 291-305.
[2] P. K. O'Neil, S. E. Rollauer, N. Noinaj, S. K. Buchanan, *Biochemistry* **2015**, *54*, 6303-6311.
